# Infrequent cross-transmission of *Shigella flexneri* 2a strains among villages of a mountainous township in Taiwan with endemic shigellosis

**DOI:** 10.1186/1471-2334-13-354

**Published:** 2013-07-30

**Authors:** Ching-Fen Ko, Nien-Tsung Lin, Chien-Shun Chiou, Li-Yu Wang, Ming-Ching Liu, Chiou-Ying Yang, Yeong-Sheng Lee

**Affiliations:** 1 Institute of Medical Sciences, Tzu Chi University, No. 701, Zhongyang Rd., Sec. 3, Hualien 97004, Taiwan; 2 The Third Branch Office, Centers for Disease Control, No. 20, Wenxin S. 3rd Rd., Taichung 40855, Taiwan; 3 Master Program, Microbiology, Immunology, and Biochemistry, School of Medicine, Tzu Chi University, No. 701, Zhongyang Rd., Sec. 3, Hualien 97004, Taiwan; 4 The Central Region Laboratory, Centers for Research, and Diagnostics, Centers for Disease Control, No. 20, Wenxin S. 3rd Rd., Taichung 40855, Taiwan; 5 Department of Medicine, Mackay Medical College, No. 46, Sec. 3, Zhongzheng Rd., Sanzhi Dist., New Taipei City 25245, Taiwan; 6 The Sixth Branch Office, Centers for Disease Control, No. 202, Sinsing Rd, Hualien 97058, Taiwan; 7 Institute of Molecular Biology, National Chung Hsing University, No. 250, Kuo Kuang Rd., Taichung 40227, Taiwan; 8 Department of Public Health, Tzu Chi University, No. 701, Zhongyang Rd., Sec. 3, Hualien 97004, Taiwan; 9 The Fourth Branch Office, Centers for Disease Control, No. 752, Sec. 2, Datong Rd., Tainan 70256, Taiwan

**Keywords:** *Shigella*, Molecular epidemiology, Disease transmission/control, Pulsed-field gel electrophoresis (PFGE), Antibiotic resistance

## Abstract

**Background:**

Shigellosis is rare in Taiwan, with an average annual incidence rate of 1.68 cases per 100,000 persons in 2000–2007. However, the incidence rate for a mountainous township in eastern Taiwan, Zhuoxi, is 60.2 times the average rate for the entire country. Traveling between Zhuoxi’s 6 villages (V1–V6) is inconvenient. Disease transmission among the villages/tribes with endemic shigellosis was investigated in this study.

**Methods:**

Demographic data were collected in 2000–2010 for epidemiological investigation. Thirty-eight *Shigella flexneri* 2a isolates were subjected to pulsed-field gel electrophoresis (PFGE) genotyping and antimicrobial susceptibility testing (AST).

**Results:**

Fifty-five shigellosis cases were identified in 2000–2007, of which 38 were caused by *S*. *flexneri* 2a from 2000–2007, 16 cases were caused by *S*. *sonnei* from 2000–2003, and 1 case was caused by *S*. *flexneri* 3b in 2006. *S*. *flexneri* 2a caused infections in 4 of the 6 villages of Zhuoxi Township, showing the highest prevalence in villages V2 and V5. PFGE genotyping categorized the 38 *S*. *flexneri* 2a isolates into 2 distinct clusters (clones), 1 and 2. AST results indicated that most isolates in cluster 1 were resistant to ampicillin, chloramphenicol, streptomycin, sulfamethoxazole and trimethoprim-sulfamethoxazole (ACSSuX); all isolates in cluster 2 were resistant to ACSSuX and tetracycline. Genotypes were primarily unique to different villages or tribes. Tribe V2-1 showed the highest endemic rates. Eighteen isolates recovered from V2-1 tribe members fell into 6 genotypes, where 5 were the same clone (cluster 1). An outbreak (OB2) in 2004 in village V2 was caused by different clonal strains; cases in tribe V2-1 were caused by 2 strains of clone 1, and those in tribe V2-2 were infected by a strain of clone 2.

**Conclusions:**

From 2000–2007, 2 *S*. *flexneri* 2a clones circulated among 4 villages/tribes in the eastern mountainous township of Zhuoxi. Genotyping data showed restricted disease transmission between the villages and tribes, which may be associated with difficulties in traveling between villages and limited contact between different ethnic aborigines. Transmission of shigellosis in this township likely occurred via person-to-person contact. The endemic disease was controlled by successful public health intervention.

## Background

Shigellosis, caused by *Shigella* species such as *Shigella dysenteriae*, *S*. *flexneri*, *S*. *boydii*, and *S*. *sonnei*, is one of the most frequent causes of diarrhea in children. Approximately 164.7 million cases are documented per year worldwide resulting in 1.1 million deaths, and two-thirds of the patients are children under 5 years of age [1]. However, these figures may be significantly underestimated [2-4]. The disease is highly contagious, with infectious doses of as low as 10–100 viable *Shigella* cells and an incubation period of 1–5 days. Large outbreaks of this disease typically occur in overcrowded areas with poor sanitary conditions or due to food or water that is contaminated by the pathogen [5-14]. Shigellosis transmission occurs primarily through person-to-person contact, most commonly among young children in households and populated institutes [15-17]. Community outbreaks have been frequently associated with daycare centers and school attendees [18,19]. Epidemic and endemic cases are most frequently caused by *S*. *dysenteriae* and *S*. *flexneri* in developing countries and *S*. *sonnei* in developed countries [1,20-23]. In the United States, 72.3% of infections are caused by *S*. *sonnei* and 14.3% are caused by *S*. *flexneri*, primarily affecting children under 9 years of age (54.1%) [21].

Shigellosis is a notifiable disease in Taiwan. In 2000–2007, 139–1,355 cases of shigellosis were identified each year, with an average incidence rate of 1.68 cases per 100,000 persons [24]. Most cases were identified in mountainous townships inhabited by aboriginal Taiwanese people, who accounts for 2% of the population in Taiwan. The incidence rate of shigellosis among aboriginal townships is 21–120 times of that in populated urban areas of Taiwan [25]. Notably, most cases observed in mountainous townships were caused by *S*. *flexneri*, whereas those in populated urban areas were caused by *S*. *sonnei*[5,6,26,27]. *S*. *flexneri* 2a was the most commonly identified agent causing endemic shigellosis among the aboriginal townships in Taiwan [5,6].

From 2000–2007, 55 shigellosis cases were identified in the villages of the mountainous township of Zhuoxi in eastern Taiwan. The township includes different ethnic aborigines living in villages/tribes. In the present study, we characterized 38 *S*. *flexneri* 2a isolates using pulsed-field gel electrophoresis (PFGE) analysis and antimicrobial susceptibility testing (AST) to investigate the mode of transmission of shigellosis among villages and tribes over a period of 8 years.

## Methods

### Epidemiological investigation

Shigellosis is one of the notifiable diseases in Taiwan; demographic information of patients and contacts are obligatory to report to Taiwan Centers for Disease Control (Taiwan CDC) by county health authorities. The epidemiological investigation of disease outbreaks was conducted by the Sixth Branch Office of Taiwan CDC and the Hualien County Health Bureau. For each shigellosis case, the patient was interviewed with a routine questionnaire to trace the possible source of infection. Information collected by the questionnaire included personal demographic information, date of onset, symptoms, travel history, source of drinking water and food, ethnicity, family and school contacts, etc. Stool specimens from contacts of patients were subjected to bacterial examination. This study was exempted from the IRB approval for using the delinked data in the Notifiable Disease Database of Taiwan CDC and not uncovering the ethnicity of the subjects.

### Bacterial isolates

From 2000–2010, stool samples of suspected shigellosis patients and their contacts in the Zhuoxi Township were taken as part of standard patient care and forwarded to the Taiwan CDC for bacteriological examination, including *Bacillus cereus*, *Escherichia coli*, *Salmonella enterica*, *Shigella spp*., *Staphylococcus aureus*, *Vibrio cholerae*, and *V*. *parahaemolyticus*. Isolates were tested by using Gram-staining, conventional biochemical tests (API 20 E test kit; bioMérieux, Marcy l’Étoile, France), and serotyping by using the slide agglutination method with commercial polyclonal antiserum (Denka Seiken, Tokyo, Japan). *Escherichia coli* ATCC 25922 and *Salmonella enterica* serovar Braenderup strain H9812 were used as reference strains for AST and PFGE analyses, respectively.

### PFGE and pattern analysis

Isolates were subjected to PFGE analysis using the standardized PulseNet PFGE protocol for *Shigella* and other enterobacteria [28], except *Not*I was used rather than *Xba*I. PFGE images were digitally recorded in tiff file format using a Kodak EDAS290 System (Eastman Kodak Co, Rochester, NY, USA). PFGE fingerprints were analyzed using BioNumerics software version 4.6 (Applied Maths; Kortrijk, Belgium). Isolates differing in 1 or more bands were considered to have different genotypes [29]. A dendrogram for the PFGE patterns was constructed using the Dice similarity coefficient and Unweighted Pair Group Method with Arithmetic Mean (UPGMA) algorithm, with settings for pattern optimization of 1.0% and band tolerance of 1.0%. In this study, isolates with PFGE pattern similarities of ≥80% were categorized in the same cluster.

### Antimicrobial susceptibility testing

Thirty-three isolates were subjected to antimicrobial susceptibility testing with 12 antimicrobials by using the microbroth dilution method with custom-designed 96-well Sensititre MIC panels (TREK Diagnostic Systems LTD., West Essex, England) following the instructions of the manufacturer. Interpretation of the MIC results followed the guidelines of the Clinical and Laboratory Standards Institute (CLSI) [30]. Twelve antimicrobial agents were tested, including ampicillin (AMP), cefotaxime (CTX), ceftazidime (CFZ), chloramphenicol (CHL), ciprofloxacin (CIP), gentamicin (GEN), imipenem (IMI), nalidixic acid (NAL), streptomycin (STR), sulfamethoxazole (SMX), tetracycline (TET), and trimethoprim-sulfamethoxazole (SXT).

## Results

### Shigellosis in Zhuoxi Township

**Figure 1:**
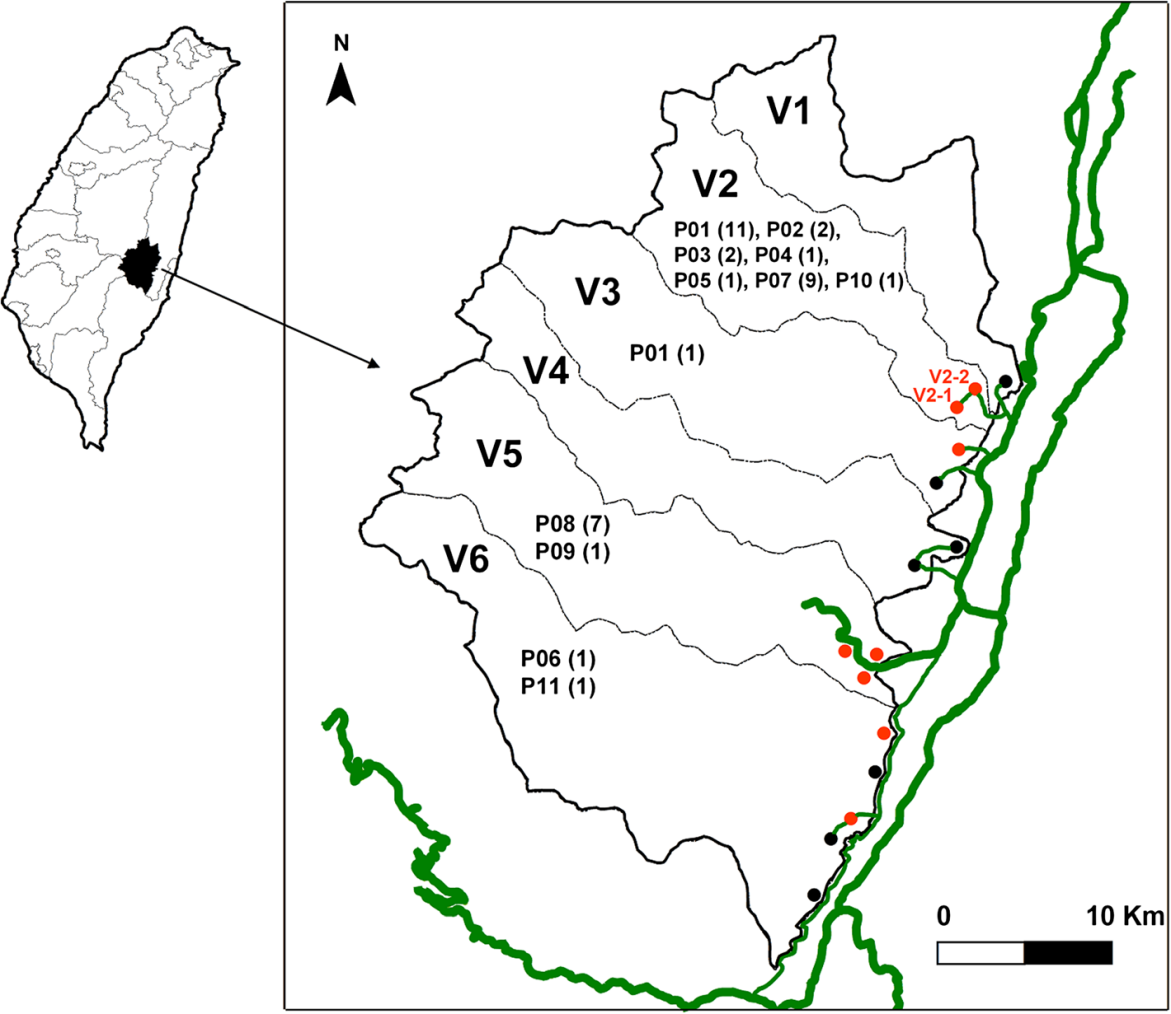
**Zhouxi Township map, showing the villages, tribes, genotypes, numbers of shigellosis cases, and road systems.** The thicker green lines represent the Provincial Highway system, while the thin green line represents a road connecting the villages and tribes. Tribes with shigellosis during 2000-2007 are indicated in red, and otherwise indicated in black. The genotypes for the isolates and cases in each village are indicated and numbered in parenthesis.

The Zhuoxi Township includes 6 villages (V1 to V6) in which 6,800 aboriginal people live. While these villages are separated by mountains, each village has a country road connected directly to Provincial Highway (Figure 1). Only trails are available for transportation between villages. V2 includes 2 tribes, V2-1 and V2-2, which contain different ethnic aborigines.

From 2000–2007, 55 shigellosis cases (an average incidence rate of 101.1 cases per 100,000 persons) were identified in 5 of the 6 villages, of which 38 cases were caused by *S*. *flexneri* 2a in 4 villages in 2000–2007, 16 cases were caused by *S*. *sonnei* in 3 villages in 2000–2002, and 1 case was caused by *S*. *flexneri* 3b in 2006 (Table 1). No shigellosis case was reported during March 2, 2007–December 31, 2010.

**Table 1:** **Distribution of shigellosis cases and incidence rates in the villages**/**tribes of Zhouxi Township**, **2000**–**2007**^**a**^

	**V1**	**V2-1**	**V2-2**	**V3**	**V5**	**V6**	**Total**	**Incidence rate (/100000)**
2000		2	5 (5)				7 (5)	98.7
2001	2 (2)	3 (1)	1 (1)	1	6 (6)		13 (10)	184.1
2002		3 (1)				1	4 (1)	57.7
2003		4					4	58.8
2004		4	9		4		17	253.4
2005		2			3	1	6	89.9
2006				1 **(1)**	1		2 (1)	30.2
2007		2^b^					2	30.7
**Total**	**2 (2)**	**20 (2)**	**15 (6)**	**2 (1)**	**14 (6)**	**2**	**55 (17)**	**101.1**

^a^Thirty eight cases were caused by *Shigella flexneri* 2a; number in parenthesis indicates the number of cases caused by *S*. *sonnei* but the case in village V3 in 2006 was caused by *S*. *flexneri* 3b. ^b^The onset date of the last shigellosis case was March 1, 2007; no shigellosis case was reported from March 2, 2007 to December 31, 2010.

Because *S*. *flexneri* 2a infections occurred widely in 4 villages and persisted for 8 years, isolates were collected for further characterization to investigate the mode of transmission among the villages. A total of 38 *S*. *flexneri* 2a isolates were recovered from 24 sporadic cases, SP1–SP24, and 2 outbreaks, OB1 (4 cases) and OB2 (10 cases) (Table 2). The median age of the patients was 7.5 years (range, 1–84), 29 of whom were aged less 13 years, while 4 were aged over 60 years.

**Table 2:** **Epidemiological information and characterization of 38 *Shigella flexneri *2a from Zhuoxi Township**^**a**^, **2000**–**2007**

**Patient no.**	**Isolate code**	**Gender**	**Age**	**Onset date (m/d/y)**	**PFGE subtype**	**Genetic cluster**	**Antibiogram**^**b**^	**Village**^**c**^	**Institution**^**d**^	**Source**^**e**^
1	E03.1201	M	1	11/17/03	P01	1	ACSSuX	V2-1	N	OB1
2	E03.1260	M	15	11/17/03	P01	1	ACSSuX	V2-1	N	OB1
3	E03.1437	F	12	11/19/03	P01	1	ACSSuX	V2-1	B	OB1
4	E03.1440	F	7	11/27/03	P01	1	ACSSuX	V2-1	B	OB1
5	E04.0408	M	8	03/26/04	P01	1	ACSSuX	V2-1	B	OB2
6	E04.0453	F	3	03/29/04	P01	1	ACSSuX	V2-1	A2	OB2
7	E04.0441	F	9	03/24/04	P02	1	ACSSuTX	V2-1	B	OB2
8	E04.0297	M	4	03/12/04	P07	2	ACSSuTX	V2-2	A1	OB2
9	E04.0319	F	7	03/16/04	P07	2	ACSSuTX	V2-2	A1	OB2
10	E04.0346	M	4	03/19/04	P07	2	ACSSuTX	V2-2	A1	OB2
11	E04.0363	M	7	03/19/04	P07	2	ACSSuTX	V2-2	B	OB2
12	E04.0354	F	10	03/23/04	P07	2	ACSSuTX	V2-2	B	OB2
13	E04.0472	F	4	03/29/04	P07	2	ACSSuTX	V2-2	A1	OB2
14	E04.0507	F	10	03/31/04	P07	2	ACSSuTX	V2-2	B	OB2
15	E00.0859	F	60	09/01/00	P05	1	ACSSuX	V2-1		SP1
16	E00.0880	M	3	09/05/00	P01	1	ACSSuX	V2-1	N	SP2
17	E01.4395	M	1	09/12/01	P01	1	ACSSuX	V2-1	N	SP3
18	E01.4508	F	41	09/17/01	P10	2	ND	V2-1		SP4
19	E02.0431	F	67	02/27/02	P01	1	ACSSuX	V2-1		SP6
20	E02.1665	F	78	10/21/02	P02	1	ACSSuTX	V2-1		SP8
21	E04.0026	F	58	01/04/04	P01	1	ACSSuX	V2-1		SP9
22	E05.0245	F	9	02/24/05	P01	1	ACSSuX	V2-1	B	SP18
23	E05.0343	M	12	03/02/05	P04	1	ACSSuX	V2-1	B	SP19
24	E07.0420	F	2	02/15/07	P03	1	ACSSuX	V2-1	N	SP23
25	E07.0426	M	9	03/01/07	P03	1	ACSSuX	V2-1	B	SP24
26	E04.1157	M	3	07/20/04	P07	2	ACSSuTX	V2-2	N	SP11
27	E04.1187	F	12	07/26/04	P07	2	ACSSuTX	V2-2	B	SP12
28	E01.6570	F	5	11/10/01	P01	1	ACSSuX	V3	N	SP5
29	E04.0925	M	4	05/21/04	P08	2	ND	V5	N	SP10
30	E04.1469	M	4	09/04/04	P09	2	ACSSuTX	V5	N	SP13
31	E04.1909	M	2	12/06/04	P08	2	ND	V5	N	SP14
32	E04.1939	M	1	12/09/04	P08	2	ND	V5	N	SP15
33	E05.0004	M	7	01/04/05	P08	2	ACSSuTX	V5	N	SP16
34	E05.0005	F	8	01/05/05	P08	2	ACSSuTX	V5	N	SP17
35	E05.1303	F	84	08/08/05	P08	2	ACSSuTX	V5		SP21
36	E06.5052	M	56	10/21/06	P08	2	ND	V5		SP22
37	E02.0464	M	80	03/12/02	P11	2	ACSSuTX	V6		SP7
38	E05.0638	M	6	05/18/05	P06	1	ACSSuX	V6	N	SP20

^a^Zhuoxi, consisting of 6 villages (V1–V6), is a mountainous township in Hualien county in eastern Taiwan. ^b^Antimicrobial resistance patterns: A, ampicillin; C, chloramphenicol; S, streptomycin; Su, sulfamethoxazole; T, tetracycline; X, trimethoprim-sulfamethoxazole. ND, AST was not done.^c^V2-1 and V2-2 are 2 tribes in village V2.^d^Two day care centers were present in the V2 village (A1, A2) as well as 1 elementary school (B). N, children were not studying in A1, A2, or B schools.^e^OB, outbreak; SP, sporadic case.

Outbreak OB1 occurred in tribe V2-1 in 2003, whereas outbreak OB2 occurred in tribesV2-1 and V2-2 in 2004. During the OB2 outbreak, rectal swabs were acquired from 186 suspected patients and their contacts. Ten *S*. *flexneri* 2a were identified, which infected children with a median age of 7.0 years. Ten children were from 2 tribes; 3 were from tribe V2-1 and 7 were from tribe V2-2. Patients were found to be living in the same village, were classmates, or were playmates.

### Antimicrobial resistance

Thirty-three *S*. *flexneri* 2a isolates were subjected to AST. Two resistance patterns were observed; 17 isolates were resistant to ACSSuX (AMP, CHL, STR, SMX, and SXT) and 16 were resistant to ACSSuTX (AMP, CHL, STR, SMX, TET, and SXT) (Table 2).

### PFGE analysis

**Figure 2:**
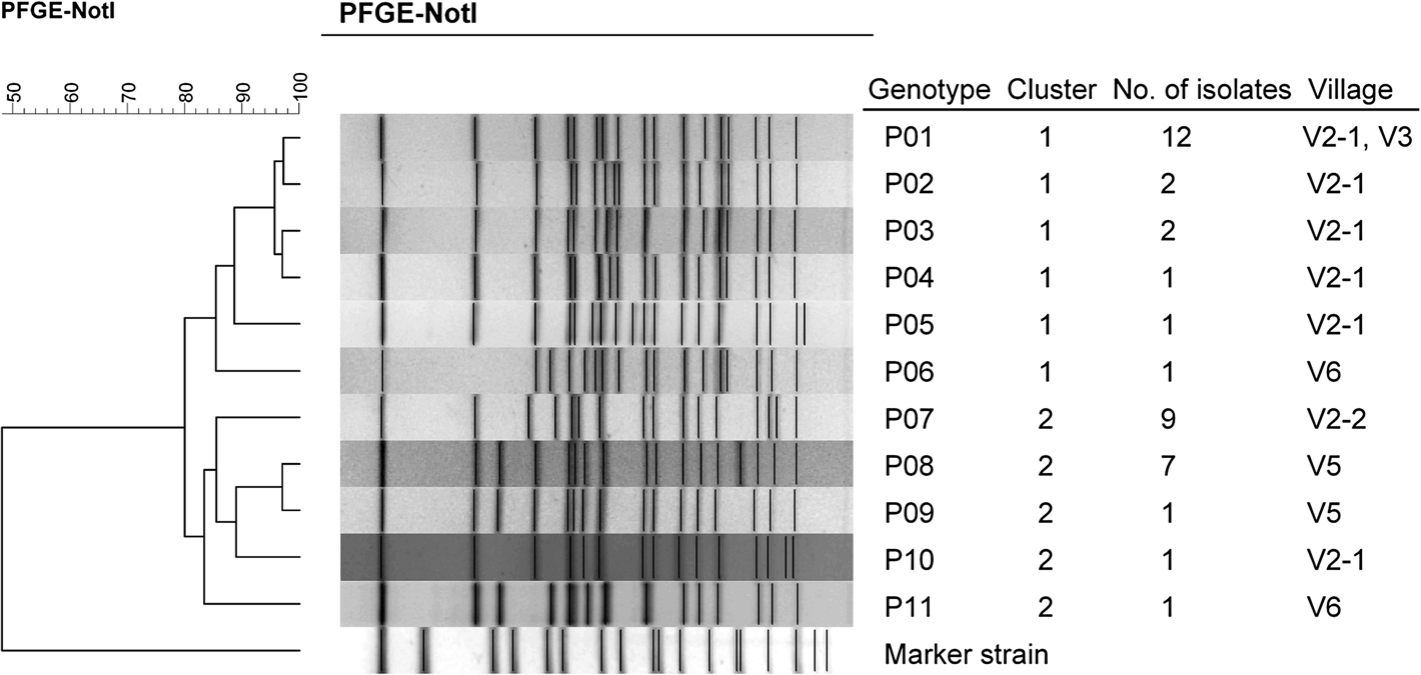
**Dendrogram of *Shigella flexneri *type 2a strains constructed based on *NotI*-pulsed-field gel electrophoresis patterns.**

All 38 *S*. *flexneri* 2a isolates recovered in 2000–2007 were subjected to PFGE analysis with *Not*I. PFGE results were used to categorize these isolates into 11 PFGE types, P01–P11. P01, P07, and P08 were most prevalent. Twelve isolates of the P01 type were responsible for 4 cases of OB1 outbreak and 2 cases of the OB2 outbreak, as well as 5 sporadic cases in tribe V2-1 and 1 case in V3 (Table 2). Nine P07 isolates were recovered from OB2 outbreak cases and 2 sporadic cases in the V2-2 tribe in 2004. Seven P08 isolates were recovered from cases in V5, which had occurred in 2004–2006. Clustering analysis of the 11 PFGE patterns revealed 2 distinct clusters, 1 and 2 (Figure 2). Strains of cluster 1 emerged in villages V2, V3, and V6, whereas strains of cluster 2 emerged in villages V2, V5, and V6. Among the 33 isolates subjected to AST, all but 2 isolates from cluster 1 displayed the ACSSuX resistance pattern, whereas all isolates from cluster 2 were resistant to ACSSuTX (Table 2). The 2 isolates showing ACSSuTX resistance in cluster 1 were of the P02 type, which displayed high pattern similarity with the P01 type. Tribe V2-1 showed endemic issues; 18 shigellosis cases caused by *S*. *flexneri* 2a occurred over a period of 8 years. The 18 isolates fell into 6 genotypes classes, of which 5 belonged to cluster 1. The isolates recovered from the outbreaks OB2 in tribe V2-1 and tribe V2-2 belonged to different genetic clusters (clones). The 8 isolates recovered in V5 fell into 2 genotypes belonging to clone 2. However, 2 isolates from V6 were different clones.

## Discussion

In Taiwan, *S*. *flexneri* and *S*. *sonnei* are the most prevalent species [5,14,31]; *S*. *dysenteriae* and *S*. *boydii* are infrequently observed, and all are found in imported cases in the most recent 2 decades [26]. *S*. *flexneri*, the predominant subserotype 2a, circulated for a long period of time in several mountainous townships in which most of the population includes Taiwanese aborigines [5,6]. *S*. *sonnei* often causes outbreaks in populated institutes, including elementary schools, military camps, jails, and daycare centers in the industrialized western region of Taiwan [26,27,31]. *S*. *sonnei* is not prevalent in aboriginal tribes and typically circulates among communities for only a short time. Since 2001, a 4-year shigellosis control program had been implemented in mountainous townships in which endemic shigellosis has been identified [25]. Currently, shigellosis is infrequently observed in aboriginal communities; it was not been detected in the Zhuoxi Township from March 2, 2007 to December 31, 2010.

*S*. *flexneri* and *S*. *sonnei* were the causes of shigellosis in the Zhuoxi Township in 2000–2007. Most infections occurred in V2 and V5. *S*. *sonnei* infections in each village lasted for only 1–2 years, whereas *S*. *flexneri* infections persisted for much longer as it circulated in tribe V2-1 for at least 8 years (Table 1). PFGE analysis of 38 *S*. *flexneri* 2a isolates revealed that nearly all villages or tribes had unique circulating strains (Table 2, Figure 1). Although tribe V2-1 had 6 genotypes, all but 1 belonged to the same clone. Two genotypes (P08 and P09) identified in isolates from V5 differed in only 1 DNA band, indicating that they were derived from a common ancestor and caused infections in the village for 3 years (from 2004–2006). Of the 11 genotypes, only P01, the most prevalent type in the V2-1 tribe, was detected in an isolate from V3 (Figure 2). These data indicate that cross-transmission of shigellosis occurred infrequently among the villages/tribes of the Zhuoxi Township during 2000–2007, which was likely due to the inconvenient transport system between villages and different ethnic groups. This reduced interaction and lowered disease transmission between geographic locations.

The OB2 outbreak occurred in tribes V2-1 and V2-2 in March 2004. The 2 tribes are composed of aborigines from different ethnic groups (Figure 1). The 10 infected children attended 2 day care centers and 1 elementary school. Two children from tribe V2-1 and 3 from tribe V2-2 attended the same elementary school (Table 2). Epidemiological investigation suggested a common infection source. However, genotyping data indicated that cases from the tribe V2-1 were infected by 2 strains (P01 and P02) of clone 1 and those from tribe V2-2 were infected by a strain (P07) of clone 2 (Table 2). Thus, OB2 was not a single source outbreak. Genotyping results and epidemiological evidence suggested that infections were likely to occur via person-to-person transmission among family members and playmates in day care center in their respective tribes rather than school where the students came from V2-1 and V2-2. For example, cases 8, 9, 10, and 13 were playmates in day care center A1 in tribe V2-2; they were infected by the same strain (genotype P07) with different onset days (Table 2), suggesting that they were transmitted via personal contact.

The 38 *S*. *flexneri* 2a isolates were discriminated by PFGE into 11 genotypes that fell into 2 genetic clusters (clones), suggesting that *S*. *flexneri* 2a had been circulating in the Zhuoxi Township for many years. Most isolates of clone 1 displayed ACSSuX resistance and were distributed in tribe V2-1 and villages V3 and V6, circulating from 2000 to 2007. All isolates of clone 2 tested belonged to the ACSSuTX resistance type, which was distributed in tribe V2-2 and the villages V5 and V6, circulating from 2004 –2005, except for 1 case identified in 2002. Although each of the 2 clones was widespread in 3 villages, disease transmission between villages is likely very rare. Briefly, in this study, clones 1 and 2 were associated with antibiogram types ACSSuX and ACSSuTX, respectively, and circulated in different geographical locations inhabited by aborigines from different ethnic groups.

Shigellosis outbreaks in schools can persist for months and can be exacerbated by the poor hygienic practices of young children, making it difficult to control [31]. Since the first case of the OB2 outbreak was reported on March 12, 2004, several intervention measures were implemented in V2 to control the disease. These measures included the following: (1) enforcement of screening of all contacts and school children by stool microbiological examination, (2) enhanced hand-washing practices and environmental disinfection in the schools and community, (3) separation of suspected cases from classrooms, dining tables, and toilets, (4) closing of day care centers for 6 days, (5) confirming shigellosis cases using cultures, (6) chlorination of the community water tower, and (7) implementation of health education of infectious diseases to the community residents. After these efforts, no shigellosis cases were reported from March 2, 2007 to December 31, 2010.

## Conclusions

*S*. *flexneri* 2a was responsible for the endemic infection in the Zhuoxi Township, which is inhabited by aborigines with different ethnic origins. Thirty-eight strains that belonged to 2 clones were identified as causing infections among the villages/tribes in 8 years. Cross-transmission of the pathogen between villages/tribes was rare, which may be due to the poor transport system between villages/tribes and different ethnic aboriginal residences. The mode of transmission of shigellosis in the township was likely via person-to-person within the communities. Since no shigellosis cases in Zhuoxi were reported from March 2, 2007 to December 31, 2010, suggesting that successful public health intervention has well controlled the endemic disease.

## Competing interests

The authors declare that they have no competing interests.

## Authors’ contributions

CFK carried out the analysis as well as wrote and revised this manuscript. YSL designed and conducted this study as well as revised this manuscript. NTL, CSC, LYW and CYY participated in the study and interpreted the study findings. MCL performed the experiments. All authors have read and approved the final manuscript.

## Pre-publication history

The pre-publication history for this paper can be accessed here:

http://www.biomedcentral.com/1471-2334/13/354/prepub
